# ﻿Characterization of the plastome of *Physaliscordata* and comparative analysis of eight species of *Physalissensu stricto*

**DOI:** 10.3897/phytokeys.210.85668

**Published:** 2022-10-05

**Authors:** Isaac Sandoval-Padilla, María del Pilar Zamora-Tavares, Eduardo Ruiz-Sánchez, Jessica Pérez-Alquicira, Ofelia Vargas-Ponce

**Affiliations:** 1 Doctorado en Ciencias en Biosistemática, Ecología y Manejo de Recursos Naturales y Agrícolas, Centro Universitario de Ciencias Biológicas y Agropecuarias, Universidad de Guadalajara, Ramón Padilla Sánchez 2100, 45200 Las Agujas, Zapopan, Jalisco, Mexico Universidad de Guadalajara Zapopan Mexico; 2 Laboratorio Nacional de Identificación y Caracterización Vegetal A(LaniVeg), Consejo Nacional de Ciencia y Tecnología (CONACyT), Universidad de Guadalajara, Ramón Padilla Sánchez 2100, 45200 Las Agujas, Zapopan, Jalisco, Mexico CONACYT Mexico City Mexico; 3 Instituto de Botánica, Departamento de Botánica y Zoología, Centro Universitario de Ciencias Biológicas y Agropecuarias, Universidad de Guadalajara, Ramón Padilla Sánchez 2100, 45200 Las Agujas, Zapopan, Jalisco, Mexico Universidad de Guadalajara Zapopan Mexico; 4 CONACYT, Mexico City, Mexico CONACYT Mexico City Mexico

**Keywords:** Boundaries, cpDNA, expansion, phylogeny, positive selection

## Abstract

In this study, we sequenced, assembled, and annotated the plastome of *Physaliscordata* Mill. and compared it with seven species of the genus *Physalissensu stricto.* Sequencing, annotating, and comparing plastomes allow us to understand the evolutionary mechanisms associated with physiological functions, select possible molecular markers, and identify the types of selection that have acted in different regions of the genome. The plastome of *P.cordata* is 157,000 bp long and presents the typical quadripartite structure with a large single-copy (LSC) region of 87,267 bp and a small single-copy (SSC) region of 18,501 bp, which are separated by two inverted repeat (IRs) regions of 25,616 bp each. These values are similar to those found in the other species, except for *P.angulata* L. and *P.pruinosa* L., which presented an expansion of the LSC region and a contraction of the IR regions. The plastome in all *Physalis* species studied shows variation in the boundary of the regions with three distinct types, the percentage of the sequence identity between coding and non-coding regions, and the number of repetitive regions and microsatellites. Four genes and 10 intergenic regions show promise as molecular markers and eight genes were under positive selection. The maximum likelihood analysis showed that the plastome is a good source of information for phylogenetic inference in the genus, given the high support values and absence of polytomies. In the *Physalis* plastomes analyzed here, the differences found, the positive selection of genes, and the phylogenetic relationships do not show trends that correspond to the biological or ecological characteristics of the species studied.

## ﻿Introduction

*Physalis* L. (Solanaceae) includes 95 morphologically and ecologically variable species (POWO 2022). The species can be annual herbs, perennial; and shrubs or arborescent perennial rhizomatous geophytes (Martínez 1998). The flowers are usually solitary, only *P.aggregata* Waterf. develops 1–3 flowers closely distributed along a short rachis and two shrub species have 1–5 flowers in axillary fascicles (*P.arborescens* L. and *P.melanocystis* Bitter). The corolla is commonly yellow but can vary to greenish, whitish, orange (*P.campanula* Standl. & Steyerm.) or purple (e.g., *P.purpurea* Wiggins and *P.solanaceus* (Schltdl.) Axelius). The fruits are green, yellow, orange, or purple berries, and are covered by an accrescent fruiting calyx (Vargas-Ponce et al. 2003; Pretz and Deanna 2020). *Physalis* is distributed naturally in the Americas and has been widely introduced in Asia and Europe (Martínez et al. 2017; Feng et al. 2020; Vdovenko et al. 2021). Some species, both annuals and perennials, grow only in restricted areas under particular environmental conditions. In contrast, other species, mostly annuals, have a wide distribution and are found in tropical habitats with varied ecological conditions (Vargas-Ponce et al. 2003; Martínez et al. 2017). *Physalis* inhabits areas from sea level to more than 3,000 m elevation, areas that have high environmental humidity levels through to deserts, with variable temperature and light conditions, in conserved environments, and with anthropocentric disturbances (Martínez and Hernández 1999; Vargas-Ponce et al. 2003, 2016; Toledo 2013). The morphological and ecological diversity of this genus is considered to be the result of different selective pressures and the independent evolutionary dynamics of each species.

*Physalis* contains species of economic, nutritional, and medicinal importance. The fruits of some species are edible and contain vitamins, minerals, carotenoids, phytosterols, and phenolic compounds that have nutraceutical and antioxidant properties (Puente et al. 2011; Valdivia-Mares et al. 2016; Shenstone et al. 2020). This genus is associated with agroecosystems and monocultures. Only four species are commonly cultivated: *P.grisea* (Waterf.) M.Martínez in the United States, *P.angulata* L. and *P.philadelphica* Lam. in Mexico, and *P.peruviana* L. in South America (Zamora-Tavares et al. 2015; Vargas-Ponce et al. 2016). Some species, such as *P.cordata* Mill., *P.minima* L., *P.pruinosa* L., and *P.pubescens* L., are traditionally used from the wild as food and medicine (Santiaguillo and Blas 2009; Kindscher et al. 2012; Taylor et al. 2012). In addition to nutritional contributions, species of *Physalis* have compounds of pharmacological interest (e.g., flavonoids, physalins, saponins, and withanolides) with antimicrobial, cytotoxic (anticancer and antitumor), neuropsychiatric, and metabolic properties (Rengifo-Salgado and Vargas-Arana 2013; Reyes-Reyes et al. 2013; Shah and Singh-Bora 2019). This diversity of metabolites potentially reflects the variability at the genetic level among species.

Chloroplasts possess photosynthetic machinery for the transformation of solar energy into chemical energy. They present their own genome, the plastome, which in spermatophytes tends to be between 120 and 180 kb long. Its circular structure consists of a large single-copy (LSC) region and a small single-copy (SSC) region separated by two inverted repeat regions (IRa and IRb), and the order and content of genes and introns are overall conserved (Daniell et al. 2016; Shetty et al. 2016; Shen et al. 2020). The proteins encoded by genes in the plastome have photosynthesis as a key function and participate in the synthesis of amino acids, fatty acids, phytohormones, and vitamins and in the assimilation of sulfur and nitrogen. In addition, they intervene in response mechanisms to unfavorable environmental conditions such as extreme temperatures, drought, and high concentrations of light and salinity (Carbonell-Caballero et al. 2015; Shen et al. 2020; Xu and Wang 2021). The plastome has been an important part of the evolutionary and adaptive process of plants.

Comparative plastomic analyses contribute to understanding the evolutionary history of different groups of plants. These comparisons help to identify whether the evolution of a particular group has occurred in parallel, presenting similar evolutionary patterns when homology among genomes is high or has occurred independently showing reticulated evolution (Carbonell-Caballero et al. 2015; D’Agostino et al. 2018; Do et al. 2020; Wu et al. 2021; Yang et al. 2021a). Plastome analysis across all photosynthetic organisms has shown that the size and number of coding DNA sequences (CDSs) are larger in algae and smaller in gymnosperms, relative to angiosperms. However, the loss of regions, genes, and introns is recurrent in all plant lineages (Mohanta et al. 2020). Additionally, pseudogenization and intron loss have been documented at lower taxonomic levels (Saxifragaceae, Liu et al. 2020); translocation, inversion, pseudogenization, or loss of genes (Opuntioideae Burnett, Köhler et al. 2020) and inverted repeat (IR) contractions (*Valeriana* L., Kim and Kim 2021) have also been observed. In contrast, some groups exhibit a high level of structural conservation and gene order and content. The variation is given by InDels (insertions and deletions) and SNPs (single nucleotide polymorphisms), as has been documented in Moraceae (Achakkagari et al. 2020), and *Artocarpus* J.R.Forst. & G.Forst. (Souza et al. 2020). Therefore, there is no single pattern that characterizes the general evolution of the plastome in spermatophytes.

Several comparative plastomic analysis have been conducted on the family Solanaceae, but for *Physalis*, few studies of the chloroplast genome have been undertaken. Feng et al. (2020) analyzed the plastome of five taxa (*P.angulata*, *P.minima*, *P.peruviana*, *P.pubescens*, and *P.alkekengi* L. (= *Alkekengiofficinarum* Moench, a genus segregated from *Physalis*). In this study, variation was seen in expansions and contractions in IRs, intergenic spacers, and nucleotide content. Sandoval-Padilla et al. (2022) compared the plastome of two samples of *P.philadelphica*, one representing the wild gene pool and the other the domesticated gene pool and found differences in microsatellite and InDels in coding and non-coding regions, with no apparent trace of changes due to the domestication process. To increase knowledge about the evolution of the plastome in the genus, we selected *P.cordata* – an annual, wild species that grows in tropical areas, and whose fruits are consumed by traditional farmers – to sequence and annotate its plastome and compare it with those of other species of *Physalis*. Our objectives were (1) to obtain and characterize the plastome of *P.cordata*, (2) to compare its structure and genetic composition with those of seven available *Physalis* plastomes, (3) to identify genes with greater variation as potential markers for genetic studies and genes that are under positive selection, and (4) to obtain a phylogenetic perspective for the genus based on the eight *Physalis* species for which whole plastome sequences exist.

## ﻿Materials and methods

### ﻿Plant material, cpDNA extraction, and sequencing

Fresh leaves of *P.cordata* were collected in the field and immediately dried with silica gel for further DNA extraction. The cpDNA was isolated based on Shi et al. (2012) and stored at the Laboratorio Nacional de Identificación y Caracterización Vegetal (LaniVeg) at the University of Guadalajara (voucher JS571, Table 1). DNA quality was assessed by spectrophotometry in a NanoDrop 2000 (Thermo Fisher Scientific). DNA integrity was determined by electrophoresis in a 1% agarose gel, and DNA quantity was analyzed by fluorometry in a Qubit 2.0 (Thermo Fisher Scientific). The sample was sequenced using the Ion Torrent platform following the manufacturer’s protocol. The cpDNA was fragmented by sonication and used to prepare the library following the standard Ion Torrent Personal Genome Machine (PGM) protocol (200 bp fragments). The library was quantified by qPCR. The template was amplified in Ion OneTouch2 and enriched in OneTouch2 ES. Sequencing was performed using the Ion PGM Hi Q View Sequencing Kit. Raw data are available under the BioProject number PRJNA870909 in NCBI.

**Table 1.** Data of *Physalis* species and *Alkekengiofficinarum* studied.

Species	GenBank accession	Reference	Voucher specimen or DNA number
*P.angulata*	MH019241	Unpublished	Not available
*P.cordata*	ON018728	This study	JS571
*P.chenopodiifolia*	MN508249	Zamora-Tavares et al. 2020	OVP539-5112011
*P.minima*	MH045577	Feng et al. 2020	PHZ3003
*P.peruviana*	MH019242	Unpublished	Not available
*P.philadelphica*	MN192191	Sandoval-Padilla et al. 2019	021118ISP
*P.pruinosa*	MH019243	Unpublished	Not available
*P.pubescens*	MH045576	Feng et al. 2020	PHZ2001
*A.officinarum*	MH045575	Feng et al. 2020	PHZ4001

### ﻿Plastome assembly and annotation

The quality of the raw reads was evaluated in FastQC 0.11.7 (Andrews 2010). Removal of low-quality reads was based on the Phred parameter (> 20) in Trimmomatic (Bolger et al. 2014). Reads were mapped to the plastome of *P.philadelphica* (Table 1) to exclude reads of nuclear and mitochondrial origin in Bowtie2 2 2.3.5 (Langmead and Salzberg 2012). Putative plastome reads were assembled de novo with SPAdes (Bankevich et al. 2012). Plastome coverage and assembly quality were performed in Quast (Gurevich et al. 2013). The complete plastome sequence was manually evaluated and corrected with IGV 2.5.0 (Thorvaldsdóttir et al. 2013). Automated annotation was performed in GeSeq (Tillich et al. 2017). tRNA genes were confirmed with tRNAscan-SE (Chan and Lowe 2019) and the remaining using BLAST in GenBank. The circular representation of the plastome was obtained in OGDraw 1.3.1 (Greiner et al. 2019).

### ﻿Comparative plastomic analysis and nucleotide variation

The complete sequence of the plastome of *P.cordata* was compared with the plastomes of seven *Physalis* species: *P.angulata*, *P.chenopodiifolia*, *P.minima*, *P.peruviana*, *P.philadelphica*, *P.pruinosa* L., and *P.pubescens*. The cpDNA of *P.chenopodiifolia* and *P.philadelphica* were stored in the LaniVeg. Accession numbers, references and voucher or DNA number of *Physalis* species are listed in Table 1. The comparison included the genome sequence total and each region’s size, gene number and functional classification, nucleotide content, and number and size of introns.

The sequences of the eight plastomes were aligned in MAFFT (Katoh and Standley 2013) for various analysis. Sequence identity between coding and non-coding regions was assessed in mVista (Frazer et al. 2004) using Shuffle-LAGAN mode without modifying the pre-established values of the remaining parameters and using *P.cordata* as a reference. The limits of the LSC/IRs and SSC/IRs regions of the eight *Physalis* plastomes and *A.officinarum* (MH045575) were evaluated in IRscope (Amiryousefi et al. 2018) using the “Manual files” option and default settings. To assess nucleotide differences between coding and intergenic regions, nucleotide diversity (π) was calculated using DnaSP v. 6.12.03 (Rozas et al. 2017).

### ﻿Characterization of repeat sequences and microsatellites

Forward, reverse, and palindromic repeat sequences in the plastomes were identified in REPuter (Kurtz et al. 2001) under the parameters of repeat unit (RU) length ≥ 21 bp, repeat identity ≥ 90%, and a Hamming distance of two. In addition, microsatellites present in each of the eight plastomes were identified with the MIcroSAtellite (MISA) identification tool (Beier et al. 2017). The search parameters were at least 10 RUs for mononucleotides, six for dinucleotides, and five for tri-, tetra-, penta-, and hexanucleotides.

### ﻿Gene selection analysis

To investigate the type of selection that has acted on *Physalis* plastome genes, we calculated the ratio of non-synonymous (Ka) and synonymous (Ks) substitutions. The Ka/Ks ratios of 51 genes that showed variation were evaluated. The aligned sequences were analyzed in KaKs_Calculator 2.0 (Wang et al. 2010). The 11^th^ genetic code (-c 11) was used. Ka/Ks ratios > 1, Ka/Ks = 1, and Ka/Ks < 1 suggested positive, neutral, and purifying selection, respectively.

### ﻿Phylogenetic analysis

To obtain a phylogenetic perspective on the relationships of *P.cordata* and the other seven species of *Physalissensu stricto* we used *A.officinarum* as outgroup. The sequences of nine plastomes were aligned in MAFFT (Katoh and Standley 2013). The evolutionary model of the whole plastome dataset without partitions was estimated in jModelTest 2.1.10 (Darriba et al. 2012). GTR + I + G was the best evolutionary model. Finally, a maximum likelihood (ML) analysis was conducted in Garli 2.01 (Bazinet et al. 2014) with 1,000 bootstrap replicates.

## ﻿Results

### ﻿Characteristics of the plastome of *Physaliscordata*

**Figure 1.**
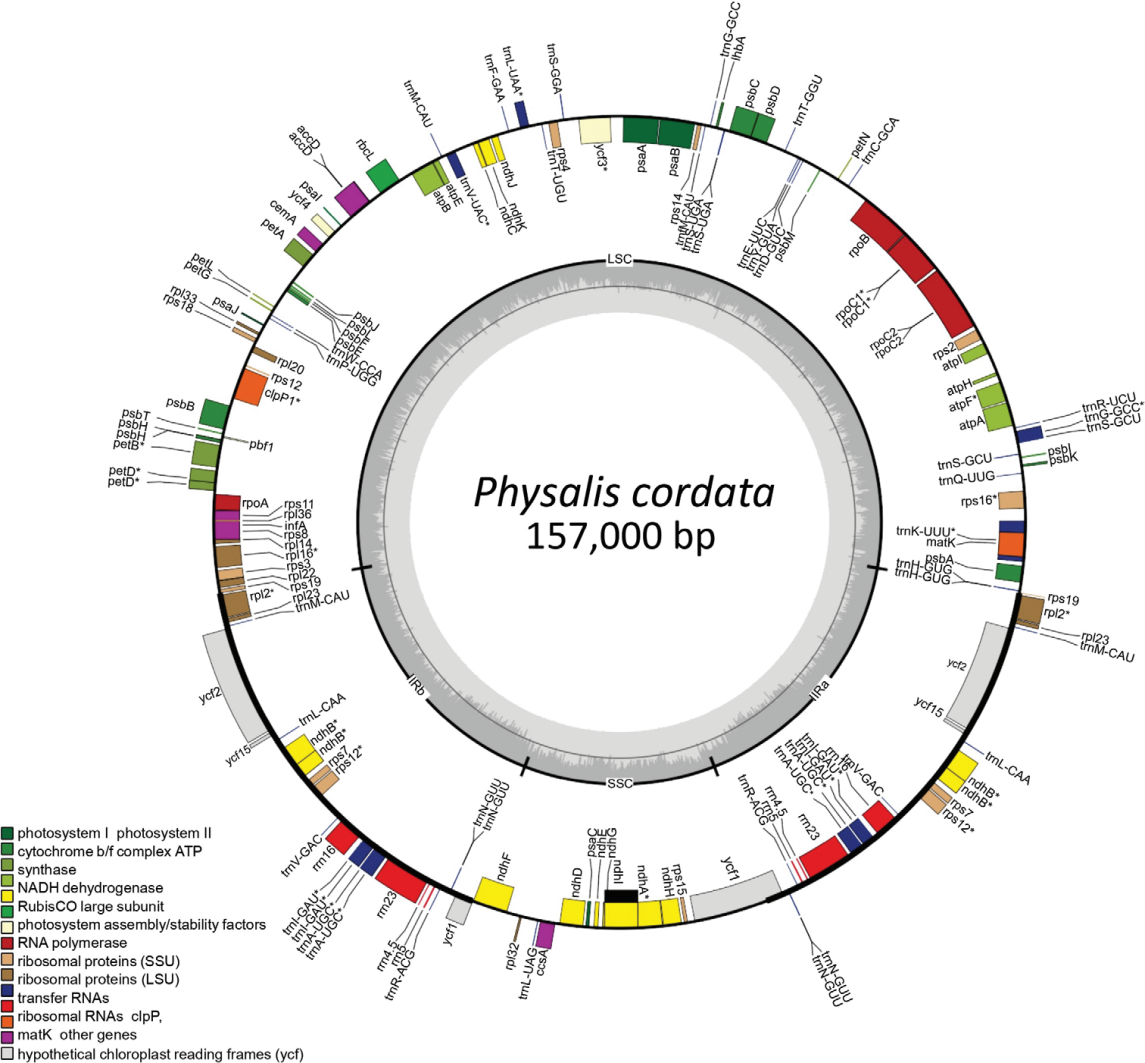
Plastome map of *Physaliscordata*. Genes located outside the outer circle are transcribed in the clockwise direction, whereas genes within the circle are transcribed in the counterclockwise direction. Genes with introns were marked with (*). Genes belonging to different functional groups are color-coded. Darker gray dashed area in the inner circle indicates GC content while lighter gray corresponds to the AT content of the plastome.

The *Physaliscordata* plastome is 157,000 bp long and presents a quadripartite structure, with an LSC region of 87,267 bp, an SSC region of 18,501 bp, and two IRs of 25,616 bp (Fig. 1). The GC content was 37.52%, with a higher content in IRs (43.08%) than in the LSC (35.57%) and SSC (31.26%) regions (Table 2). There were 115 genes and five pseudogenes, including 80 genes coding for proteins, 31 for tRNA, and four for rRNA. Twenty-two duplicate genes were identified in IRs. Nineteen introns were present in 17 genes, two genes with two introns (*clp*P and *ycf*3) and the remainder with one (*atp*F, *ndh*A, *ndh*B, *pet*B, *rpl*16, *rpl*2, *rps*12, *rpo*C1, *rps*16, *trn*A-UGC, *trn*G-GCC, *trn*I-GAU, *trn*K-UUU, *trn*L-UAA, and *trn*V-UAC). The *rps*12 gene (small ribosomal protein 12) was the only gene that was trans-spliced. This result implies that it has an intron, the first exon (5’ end) is in the LSC region, and the second (3’ end) is in IRb; therefore, it is duplicated in IRa (Table 3).

**Table 2.** Summaries of plastomes of eight *Physalis* species and *Alkekengiofficinarum*.

Characteristics		*P.angulata*	*P.cordata*	*P.chenopodiifolia*	*P.minima*	*P.peruviana*	*P.philadelphica*	*P.pruinosa*	*P.pubescens*	*A.officinarum*
Size (bp)		156,706	15,7000	15,6888	15,6692	15,6706	156,804	156,706	15,7007	156,578
LSC length (bp)		90,977	87,267	87,117	86,845	86,995	87,131	88,758	87,137	88,309
SSC length (bp)		18,395	18,501	18,451	18,503	18,393	18,483	18,394	18,500	18,363
IR length (bp)		23,667	25,616	25,660	25,672	25,695	25,595	24,777	25,685	24,953
Number of genes		114	115	113	114	114	115	114	114	115
Protein-coding genes		79	80	79	80	79	80	79	80	80
tRNA genes		31	31	30	30	31	31	31	30	31
rRNA genes		4	4	4	4	4	4	4	4	4
Genes in IR		22	22	22	22	22	22	22	22	22
Genes with introns		19	19	19	19	19	19	19	19	19
Genes in IR with introns		5	5	5	5	5	5	5	5	5
Nucleotide content	A	30.81	30.84	30.83	30.82	30.81	30.87	30.81	30.83	30.78
	C	19.1	19.07	19.08	19.09	19.08	19.06	19.1	19.09	19.14
	G	18.45	18.45	18.44	18.45	18.46	18.45	18.46	18.45	18.52
	T	31.63	31.64	31.65	31.64	31.64	31.66	31.63	31.63	31.56
GC content (%)	Total	37.55	37.52	37.52	37.54	37.54	37.51	37.56	37.54	37.65
	LSC	35.58	35.57	35.57	35.6	35.57	35.63	35.7	35.57	35.75
	SSC	31.4	31.26	31.36	31.4	31.36	31.32	31.37	31.36	31.88
	IR	43.06	43.08	43.06	43.03	43.08	43.1	43.19	43.08	42.88

bp = base pairs, LSC = large single-copy, SSC = small single-copy, IR = inverted repeat regions.

**Table 3.** Plastome gene content and functional classification in *Physalis* species.

Gene group		Gene name
**Photosynthesis**	Photosystem I	*psa*A, *psa*B, *psa*C, *psa*I, *psa*J, *ycf*3^ΨΨ^, *ycf*4
	Photosystem II	*psb*A, *psb*B, *psb*C, *psb*D, *psb*E, *psb*F, *psb*H, *psb*I, *psb*J, *psb*K, *psb*L, *psb*M, *psb*N, *psb*T, *lhb*A
	ATP synthase	*atp*A, *atp*B, *atp*E, *atp*F, *atp*H, *atp*I
	NADH dehydrogenase	*ndh*A^Ψ^, *ndh*B*^Ψ^, *ndh*C, *ndh*D, *ndh*E, *ndh*F, *ndh*G, *ndh*H, *ndh*I, *ndh*J, *ndh*K
	Cytochrome b/f complex	*pet*A, *pet*B^Ψ^, *pet*D, *pet*G, *pet*L, *pet*N
	Large subunit of RuBisCO	*rbc*L
	Large subunit of ribosome	*rpl*2*^Ψ^, *rpl*14, *rpl*16^Ψ^, *rpl*20, *rpl*22, *rpl*23*, *rpl*32, *rpl*33, *rpl*36
**Self-replication**	RNA polymerase subunits	*rpo*A, *rpo*B, *rpo*C1^Ψ^, *rpo*C2
	Small subunit of ribosome	*rps*3, *rps*4, *rps*7*, *rps*8, *rps*11, *rps*12*^Ψ^, rps12_3end, *rps*14, *rps*15, *rps*18, *rps*19
	Ribosomal RNA genes	*rrn*16*, *rrn*23*, *rrn*4.5*, *rrn*5*
	Transfer RNA genes	*trn*A-UGC*^Ψ^, *trn*C-GCA, *trn*D-GUC, *trn*E-UUC, *trn*F-GAA, *trn*fM-CAU, *trn*G-GCC^Ψ^, *trn*G-UCC, *trn*H-GUG, *trn*I-CAU*, *trn*I-GAU*^Ψ^, *trn*K-UUU^Ψ^, *trn*L-CAA*, *trn*L-UAA^Ψ^, *trn*L-UAG, *trn*M-CAU, *trn*N-GUU*, *trn*P-GGG†, *trn*P-UGG, *trn*Q-UUG, *trn*R-ACG*, *trn*R-UCU, *trn*S-GCU, *trn*S-GGA, *trn*S-UGA, *trn*T-GGU, *trn*T-UGU, *trn*V-GAC*, *trn*V-UAC^Ψ^, *trn*W-CCA, *trn*Y-GUA
**Other genes**	Hypothetical chloroplast reading frames	*orf*42*, *orf*56*, *ycf*2*, *ycf*68*, *orf*188‡
	Subunit of acetyl-CoAcarboxylase	*acc*D
	c-type cytochrome synthesis	*ccs*A
	Envelope membrane protein	*cem*A
	Protease	*clp*P^ΨΨ^
	Maturase	*mat*K
**Pseudogenes**		*inf*A, *rps*2, *rps*16^Ψ^, *ycf*1*, *ycf*15*

* Genes in IRs, ^Ψ^ genes with introns, ^ΨΨ^ genes with two introns. † only present in *P.cordata* and *P.philadelphica*, ‡ absent in *P.chenopodiifolia*.

### ﻿Comparison of the plastome of *P.cordata* with those of seven other species of *Physalis*

The comparison of the plastome of *P.cordata* with those of *P.angulata*, *P.chenopodiifolia*, *P.minima*, *P.peruviana*, *P.philadelphica*, *P.pruinosa*, and *P.pubescens* showed that all plastomes presented the typical quadripartite structure and genetic organization (Table 2). The sizes of the plastomes were variable, ranging from 156,692 bp in *P.minim*a to 157,007 bp in *P.pubescens*. The regions also varied in size; the LSC region was 86,845 bp in *P.minima* and 90,977 bp in *P.angulata*, the SSC region was 18,393 bp in *P.peruviana* and 18,503 bp in *P.minima*, and the IRs were 23,667 bp in *P.angulata* and 25,695 bp in *P.peruviana.* The total GC content was similar in all species (37.51% in *P.philadelphica* and up to 37.56% in *P.pruinosa*), and by region, the total GC content was higher in the IRs (43.03% in *P.minim* up to 43.19% in *P.pruinosa*), intermediate in the LSC region (35.57% in *P.cordata*, *P.chenopodiifolia*, *P.peruviana*, and *P.pubescens* and up to 35.7% in *P.pruinosa*), and lower in the SSC region (31.26% in *P.cordata* and up to 31.4% in *P.angulata* and *P.minima*).

The plastome of *P.cordata* presented 115 genes. This number is only shared with *P.philadelphica* since *P.angulata*, *P.minima*, *P.peruviana*, *P.pruinosa*, and *P.pubescens* have 114 genes and *P.chenopodiifolia* 113 genes. Of the species sharing 113 genes, *P.cordata* and *P.philadelphica* differed in the presence of the *trn*P-GGG gene, and *P.chenopodiifolia* was lacking *orf1*88. All species presented 22 genes in IRs and the *rps*12 gene was trans-spliced (Table 3). Of the shared genes, 103 were the same size, and 10 showed variation between species (*acc*D, *pet*B, *psb*B, *psb*C, *psb*H, *rpl*16, *rpo*C2, ψ*ycf*1, *ycf*1, *ycf*2, and *ycf*5). The number and distribution of introns were identical, 19 in 17 genes; however, 12 introns ranged from three to 99 bp (Suppl. material 1: Table S1).

### ﻿Expansion and contraction of IRs

**Figure 2.**
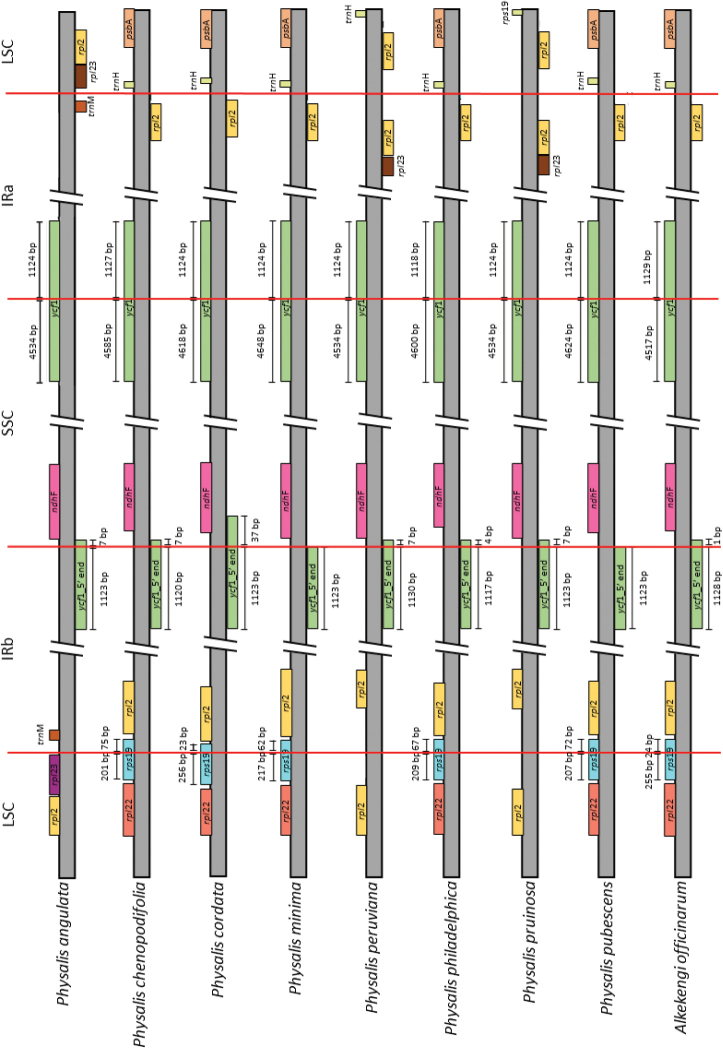
Comparison of border regions of plastomes of *Physalis* species and *Alkekengiofficinarum*.

The comparison of the limits of the LSC/IR and SSC/IR regions of the eight *Physalis* plastomes and *A.officinarum* showed some variations (Fig. 2). At the LSC/IRb boundary, the *rps*19 gene can be located at the end of the LSC region and continue at the beginning of the IRb (*P.chenopodiifolia*, *P.cordata*, *P.minima*, *P.philadelphica*, *P.pubescens*, and *A.officinarum*), presenting the second exon of *rpl*2 in the LSC region and the first in the IRb (*P.peruviana* and *P.pruinosa*) or the *rpl*23 gene in the LSC region and the *trn*M-CAU in the IRb (*P.angulata*). At the limit of IRb and the SSC region, two variations were observed: ψ*ycf*1 was in the IRb and ended at the beginning of the SSC region (*P.angulata*, *P.chenopodiifolia*, *P.cordata*, *P.peruviana*, *P.philadelphica*, *P.pruinosa*, and *A.officinarum*), followed by the *ndh*F gene or the final sequence of ψ*ycf*1, which ended at the limit of the IRb, and in the SSC region, the *ndh*F gene (*P.minima* and *P.pubescens*). In the SSC/IRa limit, the eight *Physalis* species and *A.officinarum* presented the *ycf*1 gene. Finally, the IRa/LSC limit showed three variations: the IRa may have the *trn*M-CAU gene and *rpl*23 (*P.angulata*) in the LSC region; the IRa may have the second exon of *rpl*2 and the *trn*H-GUG in the LSC region (*P.chenopodiifolia*, *P.cordata*, *P.minima*, *P.philadelphica*, *P.pruinosa*, and *A.officinarum*); or the IRa may have the first exon *rpl*2 and the second exon in the LSC region (*P.peruviana* and *P.pruinosa*) present. In addition, an extension of the LSC region and contraction in IRs were identified in *P.angulata* and *P.pruinosa*.

### ﻿Divergence in plastome sequences

**Figure 3.**
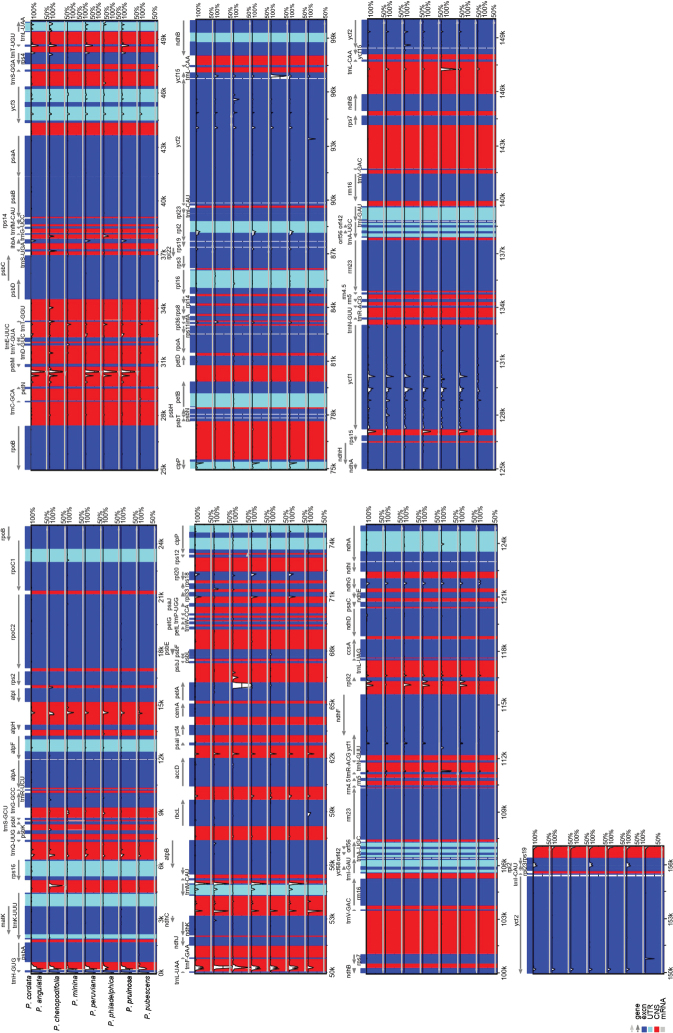
Comparative plots of identity among *Physalis* species. The percentage of identity ranges from 50 to 100% and is shown in the vertical axis. Gray arrows indicate genes with their orientation and position of their transcription in the reference plastome (*P.cordata*). Plastome regions are color coded as blue blocks for the conserved coding genes (exon), turquoise for introns and red blocks for non-coding sequences in intergenic regions (CNS).

The identity between the plastome of *P.cordata* and those of the other seven *Physalis* species was high. Identical sequences were mainly found in coding regions, and the greatest divergence was in the intergenic regions. The comparison between regions showed that the LSC and SSC regions were more divergent than were IRs. Introns also exhibited greater variation than the exons. The most divergent genes were *ycf*1 and *ycf*2, as well as the intergenic regions *trn*H-GUG-*psb*A and *trn*L-UAA-*trn*F-GAA (Fig. 3).

The sequences of 51 genes and 75 intergenic regions showed variation. The lowest variation in genes was one change in 14 genes, and the highest variation was 173 changes in *ycf*1. The lowest variation in intergenic regions was one change in 16 of them, and the highest was in *trn*L-UAA-*trn*F-GAA with 42. The average value of π was lower in the genes than in the intergenic regions (Suppl. material 1: Fig. S1). The nucleotide diversity in the genes varied from π = 0.00016 in *ndh*B to π = 0.01038 in *ycf*1 and in the intergenic regions from π = 0.0003 in *rps*7-*trn*V-GAC to π = 0.02671 in *trn*L-UAA-*trn*F-GAA. In general, 14 regions presented π values > 0.005, which included four genes (*trn*D-GUC, *trn*W-CCA, *ndh*E, and *ycf*1) and 10 intergenic regions (*trn*H-GUG-*psb*A, *trn*fM-CAU-*rps*14, *trn*L-UAA-*trn*F-GAA, *pet*A-*psb*J, *rps*18-*rpl*20, *inf*A-*rps*8, *rpl*16-*rps*3, *rpl*32-*trn*L-UAG, *trn*L-UAG-*ccs*A, and *ndh*G-*ndh*I).

### ﻿Characterization of repeat sequences and microsatellites

The repeated sequences in the plastome ranged from 35 in *P.philadelphica* to 49 in *P.cordata* (Suppl. material 1: Fig. S2). The most abundant type of repetition was forward (19 in *P.philadelphica* up to 29 in *P.cordata*), followed by palindromic (five in *P.philadelphica* up to 23 in *P.angulata*) and finally reverse (one in *P.philadelphica* up to three in *P.angulata*, *P.minima*, *P.peruviana*, and *P.pruinosa*). The number of microsatellites fluctuated from 52 in *P.peruviana* to 62 in *P.angulata* (Suppl. material 1: Fig. S3). Mono-, di-, and trinucleotide URs (repeat units) were present in all eight species; tetranucleotides were absent in *P.peruviana*, and pentanucleotides and hexanucleotides were only present in *P.angulata* and *P.pruinosa*. The types of UR with the highest number were T and A mononucleotides. In contrast, the mononucleotide C is present in a single region in five species (*P.chenopodiifolia*, *P.cordata*, *P.minima*, *P.philadelphica* and *P.pubescens*), and G was not found in any regions. In turn, the region with the highest number of microsatellites was the LSC region, followed by the SSC region, and then IRs.

### ﻿Gene selection analysis

**Figure 4.**
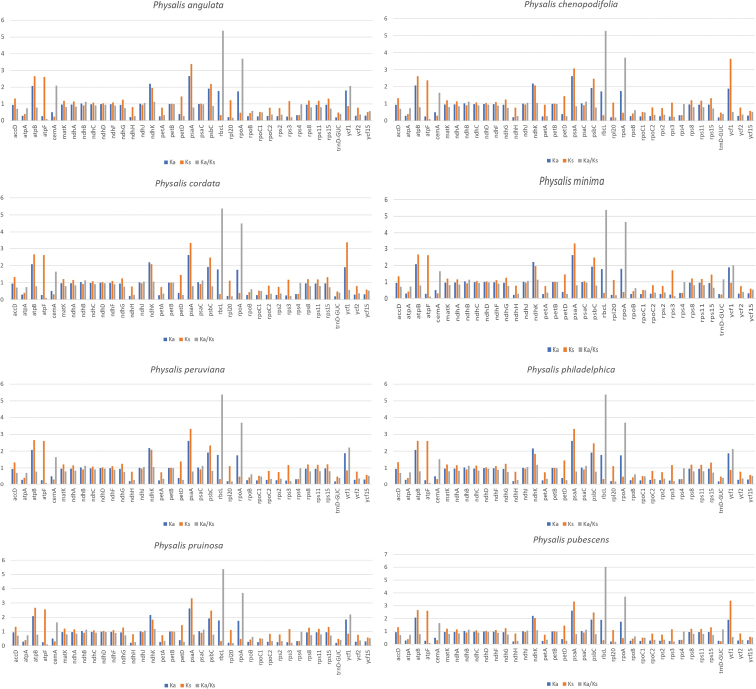
Non-synonymous substitution (Ka), synonymous substitution (Ks) and Ka/Ks values of genes of eight *Physalis* species. Blue bars indicate Ka values, orange bars indicate Ks values, and gray bars indicate Ka/Ks ratios. Genes with Ka or Ks values equal to 0 are not shown.

In 51 genes, eight showed values of Ka/Ks > 1, indicating that they are under positive selection (*cem*A, *ndh*B, *ndh*J, *ndh*K, *psa*C, *rbc*L, *rpo*A, and *ycf*1, Fig. 4). Six genes -*cem*A, *ndh*B, *ndh*J, *ndh*K, *rbc*L, and *rpo*A- are under positive selection in all eight species, *psa*C in five (*P.chenopodiifolia*, *P.cordata*, *P.peruviana*, *P.philadelphica*, and *P.pruinosa*), and *ycf*1 in four (*P.angulata*, *P.peruviana*, *P.philadelphica*, and *P.pruinosa*). The values in *ndh*B, *ndh*J, *ndh*K, and *psa*C were slightly higher than 1, compared to those in *cem*A, *rbc*L, *rpo*A, and *ycf*1, which presented higher values (Ka/Ks = 1.516 to Ka/Ks = 6.029). The other 43 genes showed Ka/Ks values <1, which indicates they are under purifying selection.

### ﻿Phylogenetic analysis

**Figure 5.**
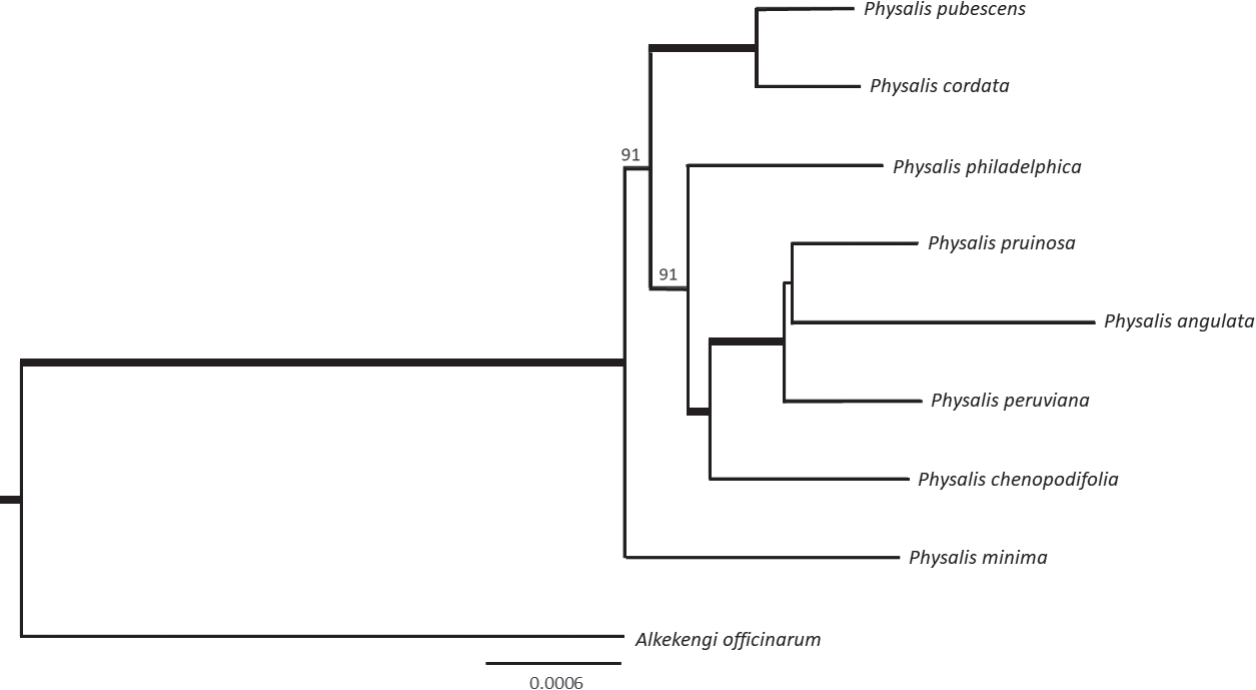
Phylogenetic tree based on Maximum Likelihood (ML) using the plastome sequence of *Physalis* species. Bold lines indicates bootstrap support values (BS) = 100, values < 100 are shown above the branches.

The ML phylogeny recovers *P.minima* as a sister to the seven other *Physalis* species included in this study (BS = 100; see Fig. 5). We recovered *P.philadelphica* as sister to the clade containing *P.pruinosa*, *P.angulata*, *P.peruviana*, and *P.chenopodiifolia* (BS = 91). This group was in turn sister to the clade formed by *P.pubescens* and *P.cordata* (BS = 91).

## ﻿Discussion

### ﻿Structure and organization of *Physalis* plastomes

The plastome of *P.cordata* analyzed here presents the typical quadripartite structure and the same order of genes as has been found for other species of the genus. However, the species vary in the total size and the size of the regions. In general, the average size of plastomes in *Physalis* is 156,814 bp, and the difference between the largest (*P.pubescens*, 157,007 bp) and the smallest plastome (*P.minima*, 156,692 bp) was 315 bp. Phylogenetically, closely related species tend to be homogeneous in size and their regions (Daniell et al. 2016; Mohanta et al. 2020). In the studied *Physalis*, however, this is true in terms of the size of the plastome but not with regions. The expansion of the LSC regions and contraction of IRs found in *P.angulata* and *P.pruinosa* is not an isolated evolutionary event; it has characterized the evolutionary history of other groups of angiosperms such as *Indigofera* L. (Fabaceae, Oyebanji et al. 2020), *Passiflora* L. (Passifloraceae, Pacheco et al. 2020), and *Corydalis* DC. (Papaveraceae, Xu and Wang 2021). Expansions and contractions in regions have occurred multiple times and in different lineages; there are models that indicate this may be due to single or double-strand breaks or be promoted by multiple inversions along with several rounds of expansions and/or contractions (Zhu et al. 2016). In *Physalis* the total GC content shows a minimal variation range of only 0.05%, ranging from 37.51% (*P.philadelphica*) to 37.56% (*P.pruinosa*). These values are similar to those documented in other genera of Solanaceae, such as *Atropa* L., *Capsicum* L., *Nicotiana* L., and *Solanum* L. (Kaur et al. 2014; Magdy et al. 2019), and families such as Asteraceae and Saxifragaceae (Zhong et al. 2019; Liu et al. 2020).

The *Physalis* species studied have between 113 to 115 genes; 113 of these are completely shared, with the same distribution and number of introns. The difference in the number of genes is based on the presence of *trn*P-GGG in *P.cordata* and *P.philadelphica* and the absence of *orf*188 in *P.chenopodiifolia*. We suggest that the genes that are not shared are the product of loss events during the evolutionary process. In addition, the size of 10 genes was different in at least one of the eight species. For example, in *P.philadelphica*, the second exon of the *pet*B gene differs by three bp with respect to those of the other species. Additionally, gene sizes are variable among the eight species, as occurs for *ycf*1, which varies from six to 114 bp. In the eight species, there were 17 genes with 19 introns, 15 genes with one intron, and two genes with two introns (*clp*P and *ycf*3). *Physalis* does not have an intron in the *pet*D gene (gene of the cytochrome b6-f subunit 4 complex), unlike that which occurs in other genera of Solanaceae such as *Atropa*, *Capsicum*, *Datura* L., *Nicotiana*, *Solanum*, and *Withania* Pauquy (Kaur et al. 2014; Mehmood et al. 2020a) and in families belonging to other orders such as Oxalidaceae (Li et al. 2021) and Lamiaceae (Zhao et al. 2020). Similarly, 12 introns presented differences ranging from three bp in *atp*F and *pet*B to 99 bp in *trn*I-GAU. In *Physalis*, the difference in the sizes of exons and introns does not impact the total size or the regions; however, changes in intergenic regions could contribute to the unequal sizes.

Variation in the boundaries of plastome regions is a relatively common evolutionary process that occurs in different plant groups (Huang et al. 2020). In the *Physalis* species studied, this variation is present in three types, distinguished by the presence of different genes at the IRs/LSC boundaries (Fig. 2). The first type is most common and it is found in *P.chenopodiifolia*, *P.cordata*, *P.minima*, *P.philadelphica*, *P.pubescens*, and *A.officinarum*. In these species the *rps*19 gene starts at LSC and ends at IRb at LSC/IRb boundary; furthermore, at IRa/LSC boundary, the second exon of the gene *rpl*2 is at IRa and the *trn*H-GUG gene at LSC. The second type is found in *P.peruviana* and *P.pruinosa*, which had the intron of the *rpl*2 gene at both of the IR/LSC boundaries. The third and most distinctive type is present in *P.angulata*, here there is change in the order of the genes, with *rpl*23 located at LSC and *trn*M-CAU at IRs (Fig. 2). The changes in *P.angulata* and *P.pruinosa* may be a product of the expansion of LSC and contraction of IRs about 2 kb (Table 2) which contrasts with the rest of the *Physalis* species analyzed, other Solanaceae genera, and several land plant families with average sizes of 25 kb in IRs (Kaur et al. 2014; Ruhlman and Jansen 2014; D’Agostino et al. 2018; Zhao et al. 2020; Yang et al. 2021a). Our results are somewhat similar to those reported by Feng et al. (2020), where the boundaries of the four regions reported in *P.angulata*, *P.minima*, *P.peruviana*, and *P.pubescens* are the same as those observed in the first type identified by us for *P.chenopodiifolia*, *P.cordata*, *P.minima*, *P.philadelphica*, and *P.pubescens*. In contrast, *A.officinarum* differs in the IRs/LSC boundaries by the presence and position of *rpl*2 gene (Fig. 2). Furthermore, this species exhibits an expansion of LSC (ca. 1.2 kb) and a contraction of IRs (ca. 0.7 kb) like that in *P.pruinosa*. Boundary variations are heritable and provide information on evolutionary and speciation processes. These mutations can be traced throughout the evolutionary process and used as evidence of shared ancestry (Stettler et al. 2021). In the *Physalis* species analyzed here there appears to be no evolutionary pattern that characterizes the boundaries of the four regions; future studies are necessary to identify a particular pattern in the genus.

### ﻿Microsatellite and repetitive regions

The variation between plastomes, in some cases, is limited due to their low rate of evolution, so repetitive regions and microsatellites can reveal interspecific variation (D’Agostino et al. 2018). In the case of repetitive regions, their divergence has been correlated to a precursor of inversions and rearrangements, so their analysis allows for different types of studies (Weng et al. 2014). In *Physalis*, repetitive regions mostly have sizes of 30–39 bp. This result coincides with those found in other genera of Solanaceae (*Nicotiana*, Mehmood et al. 2020b; and *Withania*, Mehmood et al. 2020a) and even in phylogenetically distant families such as Moraceae (Souza et al. 2020) and Poaceae (Wang et al. 2021). In turn, microsatellites have been used for the identification of plants and in analysis of population genetics and relationships between cultivars of the same species (Bassil et al. 2020). In *Physalis*, the most abundant URs are mononucleotides T and A, this may be the result of the high content of T and A in the plastome in relation to G and C. Most microsatellites are in the LSC region, which is probably because this region is longer than the SSC region and IRs. Additionally, microsatellites occur mainly in non-coding regions rather than in coding regions. The microsatellites identified in *Physalis* plastomes could be useful as potential molecular markers.

### ﻿Selection pressures

The evolutionary history of species is shaped by two main factors: mutation, which generates new genotypes, and selection, which determines the probability that new genotypes will be fixed or eliminated (Marcos and Echave 2020). If selection fixes the mutations, then the patterns of polymorphism, divergence, and gene expression are modified (Johri et al. 2020). Mutations, based on the effect of amino acid coding, are classified as Ka and Ks. Their relationship (Ka/Ks) allows us to understand the independent evolutionary history of each gene and determine if it is under positive selection (Ka/Ks > 1), purifying/stabilizing (Ka/Ks < 1), or neutral selection (Ka/Ks = 1) (Menezes et al. 2018; Yang et al. 2020a). In *Physalis*, most of the genes analyzed were under purifying selection. This implies that these regions of the plastome are maintained in terms of size and nucleotide content and that the variants that could modify the functions of the encoded proteins are eliminated (Cho et al. 2021; Yang et al. 2021a). However, eight genes were under positive selection, either in the eight species or in some of them. This result implies that certain allelic variants are fixed and benefit the optimization of physiological processes and adaptive advantages to the environment (Cho et al. 2021). Under this condition, the genes *cem*A, *ndh*B, *ndh*J, *ndh*K, *rbc*L, and *rpo*A occurred in the eight species; *psa*C occurred in five species (*P.chenopodiifolia*, *P.cordata*, *P.peruviana*, *P.philadelphica*, and *P.pruinosa*), and *ycf*1 occurred in four species (*P.angulata*, *P.peruviana*, *P.philadelphica*, and *P.pruinosa*).

Throughout the evolutionary history of the plastome, most genes have been under purifying selection due to functional limitations (Yang et al. 2020b). However, positive selection can act on those that encode proteins involved in environmental adaptive processes or during the domestication process (D’Agostino et al. 2018; Li et al. 2020b; Yang et al. 2020b). In *Physalis*, the eight genes that are under positive selection can confer certain advantages. The genes *ndh*B, *ndh*J, and *ndh*K (NADH-dehydrogenase subunits B, J, and K) protect against stress caused by high concentrations of light, stabilizing the NADH complex and adjusting the photosynthetic rate, in addition to delaying plant growth because of drought (Yang et al. 2021a). The *cem*A gene (protein that envelops the chloroplast membrane) contributes to the absorption of more CO_2_ by chloroplasts (Chen et al. 2020). The *rbc*L gene (large subunit of RuBisCO) increases the transfer of electrons during the process of photosynthesis, as well as the catalytic activity on CO_2_ (Piot et al. 2018; Gui et al. 2020). The *psa*C gene (subunit of photosystem I), which occurred in the six species that are under positive selection, increases the photosynthetic rate when plants are exposed to high concentrations of ambient light (Fischer et al. 1998). The *rpo*A gene (alpha subunit of RNA polymerase) increases the transcription and expression of plastomic photosynthetic genes so that a plant develops correctly (Mehmood et al. 2020b). Finally, the *ycf*1 gene (membrane protein) is essential for cell survival and improves the construction of the cell membrane and the importation of photosynthetic proteins that contribute to the environmental adaptation process (Ye et al. 2018; Wang et al. 2020). This gene is differentially expressed in *P.angulata*, *P.cordata*, *P.philadelphica*, and *P.pruinosa*. In contrast to that which occurs in other genera, such as in *Citrus* L. (Carbonell-Caballero et al. 2015), in *Physalis*, it is not possible to associate the differential expression of genes with biological and ecological characteristics shared between the species analyzed (Suppl. material 1: Table S2). This can be the result of the historical evolutionary process of the species, such as in the case of *ycf*1 (Carbonell-Caballero et al. 2015; Jiang et al. 2018; Yang et al. 2021b).

### ﻿Divergent regions and phylogenetic analysis

Coding and non-coding regions of plastomes both tend to have a high degree of conservation (Daniell et al. 2016; Tonti-Filippini et al. 2017). But some variable regions are routinely used in the construction of phylogenetic hypotheses, phylogeographic analysis, and population genetics (Kim et al. 2020; Li et al. 2020a; Zhang et al. 2020; Zhao et al. 2021). Our results show that the π values in the coding and non-coding regions in *Physalis* are lower than those documented in other genera of Solanaceae, such as *Nicotiana* (Mehmood et al. 2020b) and *Capsicum* (D’Agostino et al. 2018). In *Physalis*, previous phylogenetic analyses have not resolved the relationships between species due to the presence of polytomies or low support values. These studies have only included one to five of the following regions: *mat*K, *rbc*L, *ndh*F, *psb*A-*trn*H, *rpl*32-*trn*L, *trn*L-*trn*F, *trn*S-*trn*G, and *ycf*1 (Olmstead et al. 2008; Särkinen et al. 2013; Zamora-Tavares et al. 2016; Feng et al. 2018; Deanna et al. 2019). Use of more plastome regions in phylogenetic analyses has the potential to help clarify species level relationships. We recommend using regions of the plastome with values of π > 0.005 (*trn*D-GUC, *trn*W-CCA, *ndh*E, and *ycf*1 and the intergenes *trn*H-*psb*A, *trn*fM-*rps*14, *trn*L-*trn*F, *pet*A-*psb*J, *rps*18-*rpl*20, *inf*A-*rps*8, *rpl*16-*rps*3, *rpl*32-*trn*L, *trn*L-*ccs*A, and *ndh*G-*ndh*I).

The phylogenetic perspective we obtained confirms the usefulness of the plastome as a source of information for conducting phylogenetic studies in *Physalis*, despite the limited number of species studied. In comparison with other studies that include partial nucleus and chloroplast sequences (Whitson and Manos 2005; Zamora-Tavares et al. 2016; Deanna et al. 2019), our analysis had high support values and polytomies are not present. In this study, *P.minima* is rescued as a sister species to the remaining seven. This partially agrees with Deanna et al. (2019) where *P.minima* is recovered as sister to the great majority of *Physalis* species. In contrast, in the study of Whitson and Manos (2005)*P.minima* forms a clade with *P.angulata*, *P.cordata*, and *P.pubescens*. Similar to Deanna et al. (2019), in our work *P.angulata* and *P.pruinosa* maintain a sister species relationship, while in Zamora-Tavares et al. (2016)*P.pubescens* is sister to *P.angulata*. Furthermore, the phylogenetic relationship of the *Physalis* species studied based on the plastome does not reflect groupings according to the chromosome number, as *P.angulata*, *P.minima*, *P.peruviana*, and *P.pubescens* have n = 24, while the other species have n = 12. This agrees with the results of Rodríguez et al. (2021), who showed that the genera *Physalis*, *Quincula* Raf. and *Chamaesaracha* (A.Gray) Benth. & Hook comprise a lineage with asymmetric karyotypes. For its part, *A.officinarum* has a symmetric karyotype (Rodríguez et al. 2021) and is an independent lineage. Moreover, since *Physalis* includes 95 species, the inclusion of a large number of species is needed to elucidate its evolutionary history and to analyze if it has a correlation with their ecological affinities and the life history of the species.

## ﻿Conclusions

The plastome of *Physaliscordata* has the typical quadripartite structure, total size, and GC content similar with other *Physalis* species for which full plastome sequences are available. *Physalis* plastomes have 113 to 115 genes with the same distribution and number of introns. Comparative analysis among eight *Physalis* species showed differences in the boundary of the LSC/IR and SSC/IR regions and three distinct types were identified, given by the variation in genes present. The high percentage of conservation of the sequences and the variation observed at the boundaries of the plastome regions, in the *ycf*1 and *ycf*2 genes, and in some coding and intergenic regions are relatively common evolutionary processes, and is seen here in all the *Physalis* species studied. Likewise, the presence of genes under positive selection, in some or all of the *Physalis* species analyzed, suggest that they are differentially expressed, and could favor the photosynthetic process and environmental adaptation, which needs to be verified. We have shown that the plastome is potentially useful for further phylogenetic studies if key highly variable genes are used. Finally, we identified that despite the level of conservation in the plastome of *Physalis*, variation in sequence does exist and probably reflects independent evolutionary processes. Future studies should include a larger number of species representing the variation in biological and ecological characteristics to understand the evolution of the plastome in *Physalis*.

## Supplementary materials

Supplementary material 1 Tables S1, S2 and Figures S1–S3 Data type Tables and images of plastome data and atributes (MS Word file) Explanation note The tables contains data about introns in choroplast genes and biological and ecological traits of *Physalis* species included in the study. Graphs show data about type of microsatellites and frequency. https://binary.pensoft.net/file/753167This dataset is made available under the Open Database License (http://opendatacommons.org/licenses/odbl/1.0/). The Open Database License (ODbL) is a license agreement intended to allow users to freely share, modify, and use this Dataset while maintaining this same freedom for others, provided that the original source and author(s) are credited.Isaac Sandoval-Padilla, María del Pilar Zamora-Tavares, Eduardo Ruiz-Sánchez, Jessica Pérez-Alquicira, Ofelia Vargas-Ponce
