# Supplementary material for: ﻿Characterization of the plastome of Physaliscordata and comparative analysis of eight species of Physalissensu stricto
Source: PhytoKeys. 2022 Oct 5;210:109–34. doi: 10.3897/phytokeys.210.85668 (PMC9836641; doi:10.3897/phytokeys.210.85668)
Supplement: Supplementary material 1 — Tables S1, S2 and Figures S1–S3 [file phytokeys-210-109_article-85668__-s001.docx]

**Table S1.** Length of introns presents in genes of *Physalis* species.

| **Gene** | **Region** | ***P. cordata*** | ***P. angulata*** | ***P. chenopodifolia*** | ***P. minima*** | ***P. peruviana*** | ***P. philadelphica*** | ***P. pruinosa*** | ***P. pubescens*** |
| --- | --- | --- | --- | --- | --- | --- | --- | --- | --- |
| *trn*K-UUU | LSC | 2509 | 2509 | 2509 | 2509 | 2509 | 2509 | 2509 | 2509 |
| *rps*16 | LSC | 855 | 855 | 855 | 855 | 855 | 855 | 855 | 855 |
| *trn*G-GCC | LSC | 693 | 692 | 698 | 694 | 692 | 693 | 692 | 692 |
| *atp*F | LSC | 710 | 707 | 710 | 710 | 707 | 707 | 707 | 709 |
| *rpo*C1 | LSC | 719 | 719 | 719 | 724 | 719 | 719 | 719 | 719 |
| *ycf*3 | LSC | 776 | 782 | 782 | 782 | 782 | 782 | 782 | 782 |
|  |  | 737 | 743 | 743 | 738 | 743 | 738 | 743 | 736 |
| *trn*L-UAA | LSC | 495 | 500 | 507 | 495 | 500 | 468 | 500 | 495 |
| *trn*V-UAC | LSC | 569 | 569 | 569 | 569 | 569 | 569 | 569 | 569 |
| *clp*P | LSC | 639 | 638 | 638 | 640 | 638 | 638 | 638 | 635 |
|  |  | 790 | 787 | 791 | 787 | 787 | 790 | 787 | 789 |
| *pet*B | LSC | 747 | 747 | 747 | 747 | 747 | 744 | 747 | 747 |
| *rpl*16 | LSC | 959 | 959 | 959 | 962 | 959 | 959 | 959 | 952 |
| *rpl*2 | IRs | 663 | 663 | 663 | 663 | 663 | 663 | 663 | 663 |
| *ndh*B | IRs | 679 | 679 | 679 | 679 | 679 | 679 | 679 | 679 |
| *trn*I-GAU | IRs | 719 | 719 | 719 | 719 | 719 | 719 | 818 | 719 |
| *trn*A-UGC | IRs | 811 | 811 | 811 | 811 | 811 | 811 | 811 | 811 |
| *rps*12 | IRs | 536 | 536 | 536 | 536 | 536 | 536 | 536 | 536 |
| *ndh*A | SSC | 1147 | 1146 | 1146 | 1148 | 1146 | 1152 | 1146 | 1146 |

**Table S2.**Biological and ecological traits of species of *Physalis sensu lato* studied.

| **Specie** | **Habit** | **Habitat** | **Climate** | **Human management** | **Altitud** | **References** |
| --- | --- | --- | --- | --- | --- | --- |
| *Alkekengi officinarum* | Perennial | Deciduous Forests | Temperate | Managed | 800-2500 | 1 |
| *Physalis angulata* | Annual | Tropical Deciduous Forests / Pine-oak forest | Hot dry | Locally managed in Mexico | 0-2400 | 2 |
| *Physalis chenopodifolia* | Perennial | Pine-oak forest | Hot dry | Harvested | 2000-3100 | 2 |
| *Physalis cordata* | Annual | Tropical Deciduous Forests | Hot dry | Harvested | 0-1300 | 2 |
| *Physalis minima* | Annual | Tropical forest | Hot dry | Managed | 1000-1800 |  |
| *Physalis peruviana* | Perennial | Mountain regions / Temperate forest | Temperate | Cultivated | 500-3600 | 3 |
| *Physalis philadelphica* | Annual | Tropical Deciduous Forests / Pine-oak forest | Hot dry | Cultivated | 0-2300 | 2 |
| *Physalis pruinosa* | Annual | Tropical Deciduous Forests / Oak forest | Hot dry | Occasionally managed | 120-1850 | 2 |
| *Physalis pubescens* | Annual | Pine-oak forest / Antropogenic disturbance | Hot dry | Harvested | 1030-2610 | 2 |

**Note:** anthropogenic disturbance can include open areas for cultivations, where Physalis are found as weed or forest clearings where are found as ruderal.

1. Zhang, Z.Y., Lu, A.M., D’Arcy, W.G., 1994. Solanaceae. In Wu, Z.Y., Raven, P.H., [eds] Flora of China vol. 17. Missouri Botanical Garden Press, St Louis, MO.

2. Vargas, O., Martínez, M., Dávila, P., 2003. La familia Solanaceae en Jalisco -El género *Physalis*-. Universidad de Guadalajara. Guadalajara. Mexico. 126 pp.

3.Chacón, M.I., Sánchez Y.P., Barrero, L.S., 2016. Genetic structure of a Colombian cape gooseberry (*Physalis peruviana* L.) collection by means of microsatellite markers. Agron.Colomb.34, 5-16.


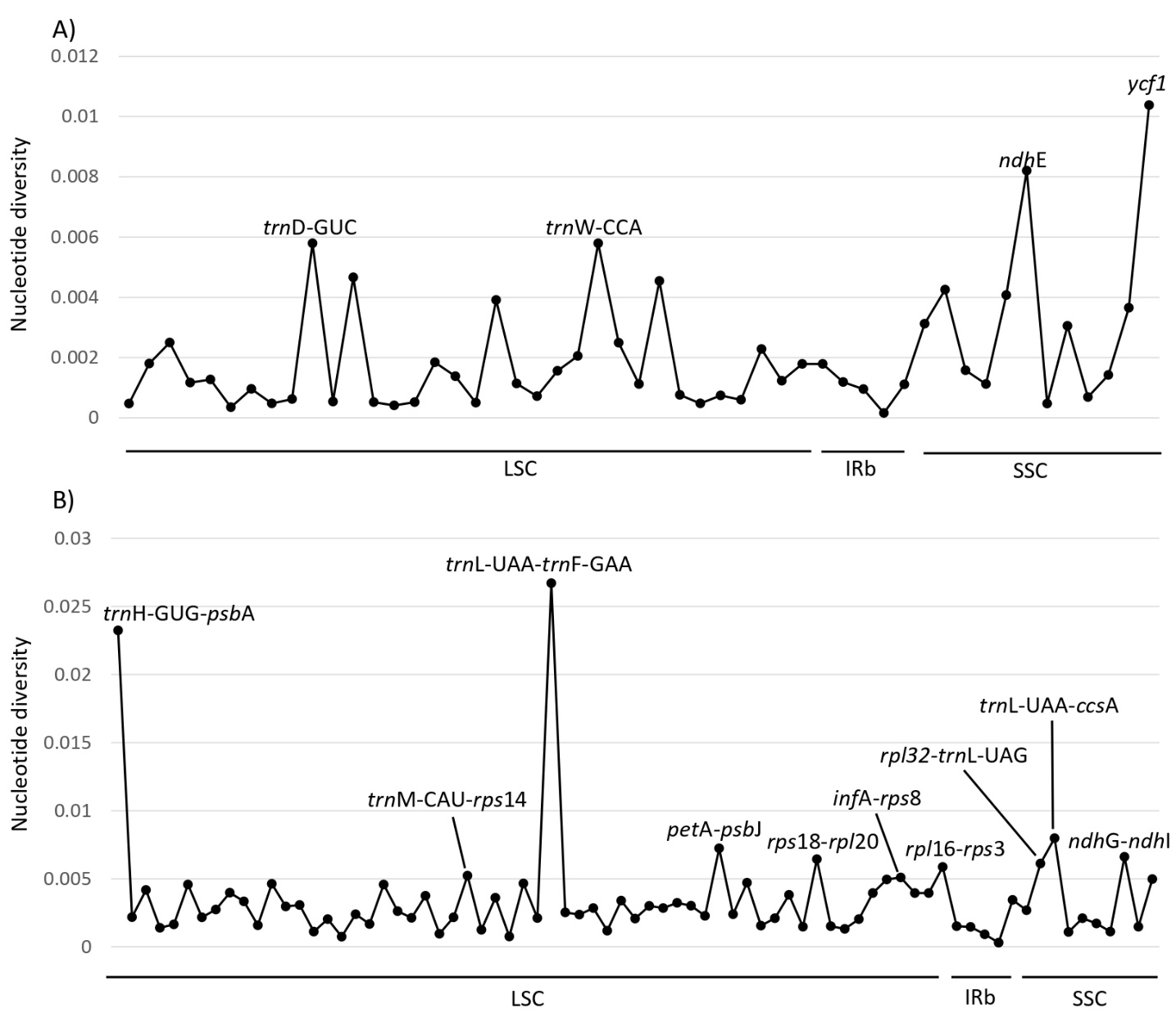
**Fig.S1**. Nucleotide diversity of coding (A) and non-coding (B) regions among *Physalis* species.


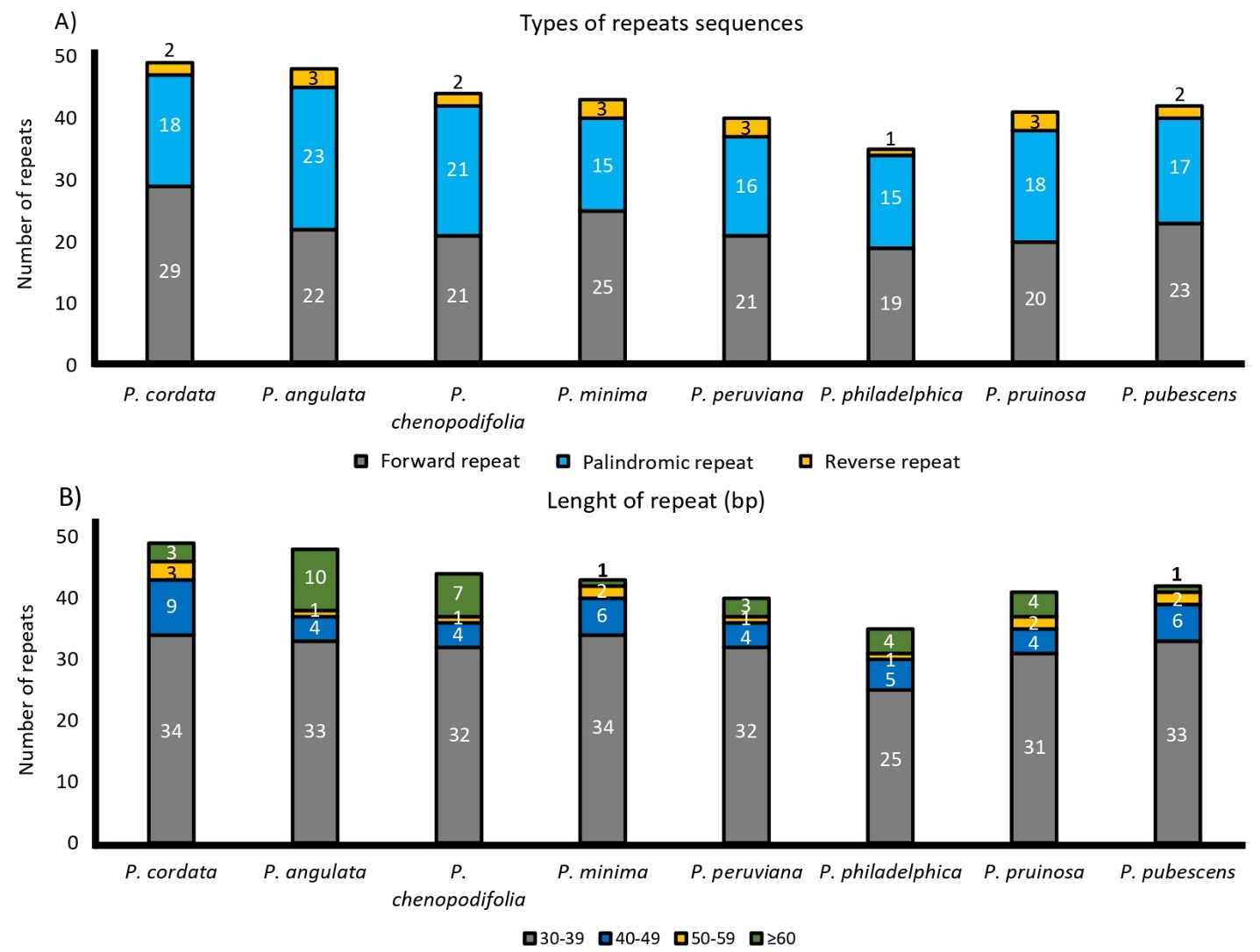


**Fig.S2**. Repeated sequences in the plastomes of the eight *Physalis* species. A) The number and types of repeated sequences. B) Number of repeated sequences per length.


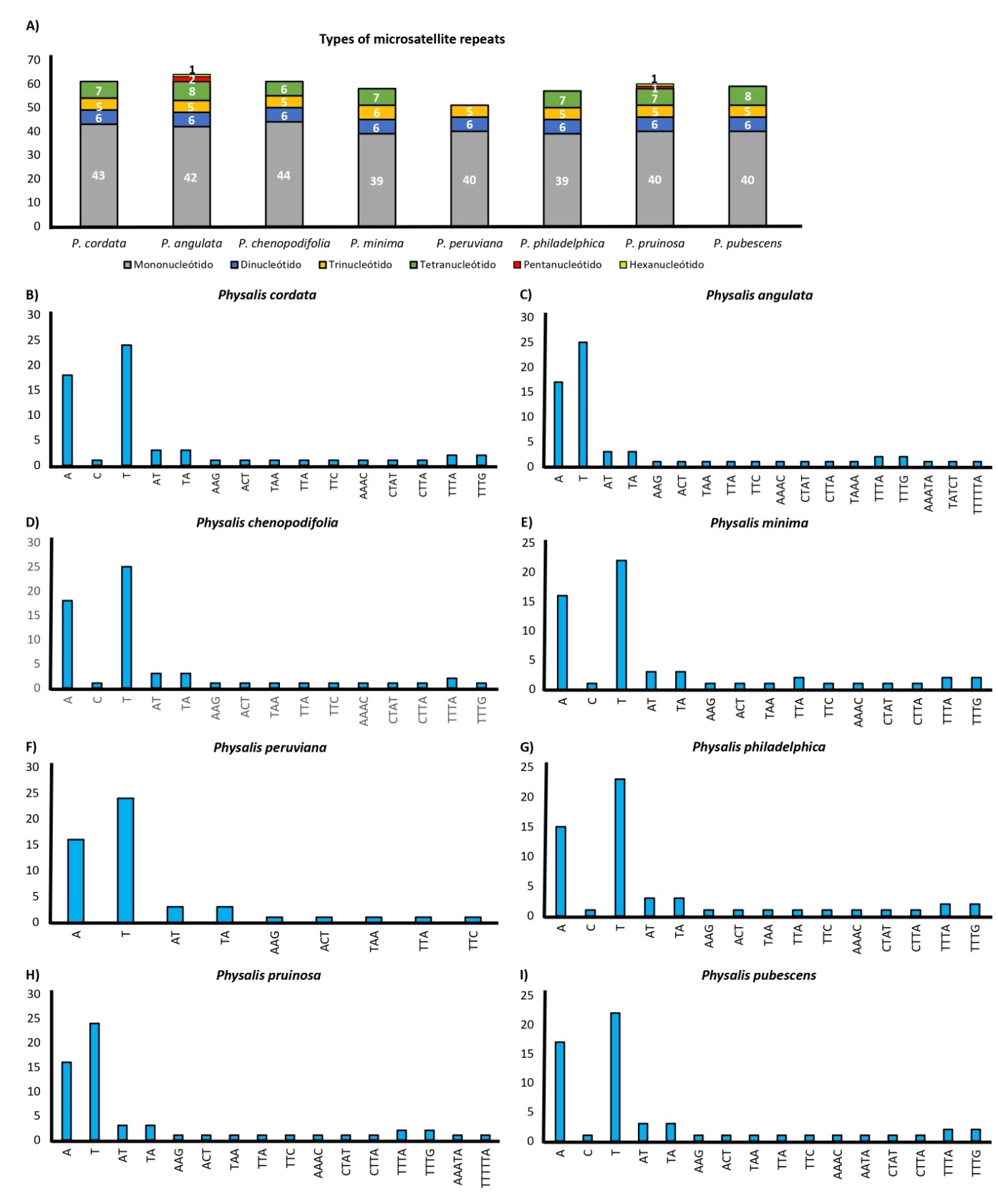


**Fig.S3**. Distribution of microsatellite regions in the eight *Physalis* plastomes. A) Number of different microsatellite types detected for each specie. B)-I) Type of repetitive units and number of microsatellite loci identified.
